# Complete Genome Sequence of *Rhodoferax* sp. Strain BAB1, Isolated after Filter Sterilization of Tap Water

**DOI:** 10.1128/MRA.00668-20

**Published:** 2020-09-17

**Authors:** Philipp Aurass, Antje Flieger

**Affiliations:** a Department of Enteropathogenic Bacteria and Legionella, Robert Koch Institute, Wernigerode, Germany; University of Southern California

## Abstract

Here, we announce the complete genome sequence of *Rhodoferax* sp. strain BAB1, which was isolated from filter-sterilized tap water. The genome consists of a 3.82-Mb chromosome. Moreover, we provide base methylation data and evidence of incomplete retention by 0.22-μm filters for this putative novel *Rhodoferax* species.

## ANNOUNCEMENT

**FIG 1:**
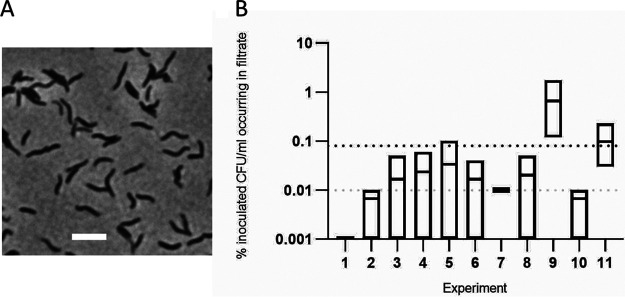
Incomplete retention of *Rhodoferax* sp. strain BAB1 by 0.2-μm filtration. (A) Image of *Rhodoferax* sp. strain BAB1 at ×1,000 magnification (Nikon Ti-E microscope), showing curved bacteria with approximate dimensions of 0.2-μm diameter and 2.5-μm length. Bar = 2.5 μm. (B) Proportions of CFU in 1 ml of filtrate, relative to 1 ml of the solution before filtration, given as percentages. Each filtration experiment was performed in triplicate. The mean penetration in all 11 experiments was 0.09% (black dotted line), with a median of 0.01% (gray dotted line). Three control filter-challenging tests using Escherichia coli DH5α and *R. saidenbachiensis* were undertaken with the same filters (Minisart, 0.2 μm; Sartorius) and the same conditions and yielded 100% retention rates.

The genus *Rhodoferax* represents rod-shaped or curved (1) Gram-negative bacteria, currently encompassing eight named species (1–8). Whereas characterized *Rhodoferax* species have been isolated from freshwater, sewage, and sediment (1, 5, 6), we repeatedly isolated strain BAB1 from previously filter-sterilized (0.22-μm pore size) tap water. The water originated from a household in Potsdam, Germany. Water samples (500 ml) were filter sterilized by means of 0.22-μm polyethersulfone filters (Steritop; Millipore) according to the manufacturer`s instructions. Presumably sterile samples were cultivated on Reasoner’s 2A (R2A) agar at 24°C for 5 days and interestingly revealed contamination in the filtrate. Successive filter-challenging tests were undertaken with bacteria grown on R2A medium. For this purpose, 19 ml phosphate-buffered saline containing 1.4 × 10^5^ CFU/ml bacteria was filtered through 0.2-μm cellulose acetate syringe filters (Minisart; Sartorius) according to the manufacturer`s instructions. Eleven independent experiments showed that the strain steadily passed through the filters at a low frequency (Fig. 1). To determine the strain’s identity, its 16S rRNA gene was amplified from lysed colony material by PCR with the universal primer pair 27f and 1525r (9) and high-fidelity DNA polymerase (Phusion; NEB). Sanger sequencing of the amplicon and comparison of its sequence with sequences of type strains by means of BLASTn (10) revealed the greatest sequence similarities to *Rhodoferax ferrireducens* strain T118 (98.17%; GenBank accession number CP000267.1), *Rhodoferax saidenbachiensis* strain DSM 22694 (98.17%; CP019239.1), *Rhodoferax sediminis* strain CHu59-6-5 (97.90%; CP035503.1), and *Rhodoferax antarcticus* DSM 24876 (97.51%; CP019240.1). The current threshold of 16S rRNA gene sequence similarity for differentiation between two species is 98.65% (11). The DNA-DNA relatedness values estimated by digital hybridization between strain BAB1 and the four close relatives identified by 16S rRNA gene sequence similarity were lower than 70% (12). These results indicate that strain BAB1 represents a putative novel species of the genus *Rhodoferax*.

For genome sequencing, genomic DNA was isolated from bacteria grown for 48 h in R2A broth at 24°C with shaking (120 rpm) with the QIAamp DNA minikit (Qiagen). Genome sequencing using a PacBio RS II sequencer (13), library preparation (SMRTbell library), quality control, raw read filtering, and genome assembly using an HGAP-based pipeline (SMRT Portal version 2.3.0, RS_HGAP_Assembly.3 protocol) were carried out by a Pacific Biosciences-certified service provider (GATC Biotech, Germany) using default parameters (14). Sequencing on two single-molecule real-time (SMRT) cells generated 118,584 reads (*N*_50_ values, 19,454 and 18,562 bp, with mean read quality control scores of 0.84 throughout). The assembly resulted in one contig representing a circular sequence, corresponding to a 3.82-Mb chromosome with 279-fold average base coverage. The ring closure was confirmed by Sanger sequencing of the amplicon obtained by PCR using high-fidelity DNA polymerase with primers binding at both ends of the contig (5′-GGACTTACGGGCATGAGTGAATCG and 5′-AAAGATCGGCGCAGCGGTGAAGAC). Sanger sequences were evaluated with Geneious version 2019.2.1 (Biomatters Ltd.).

The *Rhodoferax* sp. strain BAB1 genome has an average G+C content of 65.6%. Annotation was performed by the RAST server (version 2.0) (15) and the NCBI Prokaryotic Genome Annotation Pipeline (PGAP) (version 4.11) (16) using default parameters. Based on RAST annotation, 3,562 coding sequences were detected on the chromosome. Putative functions were assigned to 2,753 coding sequences, with 809 sequences encoding hypothetical proteins.

### Data availability.

The genome sequence and base modification data have been deposited in GenBank under the accession number CP054424 (BioProject PRJNA637161). The SRA accession number is SRR11977808.
